# Oral Anticoagulation Registry in Patients with Atrial Fibrillation Treated in Primary Care in Clinical Practice: The RACOVIR Study

**DOI:** 10.3390/jcm15218736

**Published:** 2026-11-09

**Authors:** Jose Polo-García, Rafael M. Micó-Pérez, Esperanza García-Gabriel, Juan Carlos Romero-Vigara, Antonio Segura-Fragoso, Aurora García-Lerín, Valeria Kopytina, Carlos Santos-Altozano

**Affiliations:** 1 Casar de Cáceres Health Center, Extremadura Health Service, 10190 Casar de Cáceres, Spain; jpolog@semergen.es (J.P.-G.); espe71g@yahoo.es (E.G.-G.); 2 SEMERGEN Foundation, 28009 Madrid, Spain; investigacion@semergen.org; 3 Xàtiva-Ontinyent Department of Health, Fontanars Dels Alforins Health Center, 46635 Valencia, Spain; 4 Alfajarín Health Center, Aragon Health Service, 50172 Alfajarín, Spain; jcromerovigara@hotmail.es; 5 Epidemiology Unit, SEMERGEN Research Agency, 28009 Madrid, Spain; asegurafr@gmail.com; 6 Faculty of Health Sciences, University of Castilla-La Mancha, 45600 Talavera de la Reina, Spain; 7 Almendrales Health Center, Madrid Community Health Service, 28026 Madrid, Spain; aglerin@semergen.es; 8 Suerte de Saavedra Health Center, Extremadura Health Service, 06009 Badajoz, Spain; csantosa@semergen.es

**Keywords:** atrial fibrillation, clinical practice, direct oral anticoagulants, primary care, vitamin K antagonists

## Abstract

**Objectives**: This study aims to describe real-world oral anticoagulation management in patients with NVAF followed up in Spanish Primary Care, including anticoagulant use, VKA control, treatment switching, patient-reported satisfaction, and clinical outcomes during documented anticoagulant exposure. **Methods**: RACOVIR was a multicenter observational registry with cross-sectional enrolment and retrospective collection of electronic medical record data from AF diagnosis to inclusion. Adults with NVAF receiving oral anticoagulation for ≥6 months were included. The Barthel Index and Anti-Clot Treatment Scale assessed autonomy and treatment satisfaction at inclusion. Clinical events were evaluated using exposure-based incidence rates, supplemented by weighted marginal structural models and propensity-score-adjusted Cox sensitivity analyses restricted to patients receiving a single anticoagulant strategy. **Results**: Among 1901 patients, 428 received VKAs and 1473 DOACs at enrolment. Mean exposure duration was 7.6 years for VKAs and 3.8 years for DOACs. Adequate VKA control was achieved by 56.2% and 59.0% using the direct and Rosendaal methods, respectively. Reduced DOAC doses were recorded in 24.8% of apixaban, 54.7% of dabigatran, 26.9% of edoxaban, and 21.9% of rivaroxaban users; appropriateness could not be assessed for every patient. Overall, 395 patients (20.8%) received both strategies. DOAC users reported greater treatment satisfaction. Unadjusted exposure-based incidence rate ratios (DOAC/VKA) were 1.75 (95% CI 1.05–2.92) for stroke, 1.78 (95% CI 1.06–3.00) for ischemic stroke, 1.64 (95% CI 0.97–2.79) for myocardial infarction, and 3.68 (95% CI 1.81–7.46) for major bleeding. In weighted marginal structural models, adjusted incidence rate ratios (DOAC/VKA) were 1.627 (95% CI 0.785–3.375) for stroke and 6.725 (95% CI 2.106–21.473) for major bleeding. A propensity-score-adjusted Cox sensitivity analysis excluding treatment switchers (*n* = 1492) yielded corresponding hazard ratios of 2.577 (95% CI 0.930–7.144; *p* = 0.069) and 8.298 (95% CI 3.180–21.649; *p* < 0.001), respectively. **Conclusions**: RACOVIR shows substantial heterogeneity in anticoagulation management in Spanish Primary Care. DOACs predominated and were associated with greater patient-reported satisfaction, while suboptimal INR control remained common with VKAs. Although adjusted analyses suggested higher major bleeding rates during DOAC exposure, these findings should be interpreted cautiously because of the observational design, potential selection bias, treatment switching, and residual confounding. No causal conclusions regarding comparative anticoagulant safety or effectiveness can be established.

## 1. Introduction

Atrial fibrillation (AF) is the most common sustained cardiac arrhythmia worldwide, with increasing numbers due to population ageing and the higher prevalence of cardiovascular risk factors, such as hypertension, diabetes or obesity. In fact, it has been estimated that nearly 60 million people had AF in 2023. AF is linked to substantial morbidity and mortality, including increased risks of ischemic stroke, heart failure, dementia, or cardiovascular death, and huge healthcare costs [1].

Long-term oral anticoagulation plays a central role in preventing AF-related complications. Vitamin K antagonists (VKAs) were the first anticoagulants used in patients with AF. They reduce stroke risk by about two-thirds and mortality by about one-quarter versus no anticoagulation and have been widely used worldwide with proven benefit [2]. VKA use is limited by several well-known drawbacks, including a narrow therapeutic window, multiple food and drug interactions, variable pharmacokinetics, and the need for frequent monitoring and dose adjustment [3]. In contrast, direct oral anticoagulants (DOACs) overcome many of these limitations and provide greater efficacy than VKAs for stroke prevention in AF, with lower rates of major bleeding and intracranial hemorrhage [4].

Although randomized trials support DOACs as the preferred anticoagulant option for most eligible patients with NVAF, real-world studies remain necessary because anticoagulation management in routine practice is more complex than in trial settings. In Spain, observational studies have shown variability in anticoagulant use, frequent suboptimal VKA control, and substantial heterogeneity in treatment pathways [4,5,6,7,8]. Spain has also historically had lower DOAC prescription rates than several other European countries, while Primary Care plays a major role in long-term AF management [4,5,6,7,8]. A nationwide Primary Care registry can therefore provide clinically useful information on anticoagulant use, quality of VKA control, treatment switching, patient experience, and patterns of clinical events in routine care.

The RACOVIR study aimed to describe oral anticoagulation management among patients with NVAF treated in Spanish Primary Care. Secondary objectives were to assess VKA control, characterize patients’ clinical and epidemiological profile, describe DOAC dosing and treatment switching, evaluate autonomy and treatment satisfaction, and explore thromboembolic and bleeding events during documented VKA or DOAC exposure. The registry rationale, design, and baseline characteristics have been published previously [9]. This analysis extends that work by focusing on anticoagulation quality, treatment exposure and switching, patient-reported satisfaction, and clinical events. The primary objective of this secondary report was to characterize real-world anticoagulation management and quality; clinical event comparisons were secondary exploratory analyses.

## 2. Materials and Methods

RACOVIR [9] was a multicenter observational registry conducted in Spanish Primary Care. It was not a prospective randomized comparison of anticoagulant strategies. Patients were enrolled at a study visit, and clinical and treatment data from AF diagnosis to inclusion were collected retrospectively from electronic medical records. Eligible patients were adults aged ≥18 years with documented NVAF who had received a VKA or DOAC for at least 6 months before inclusion; VKA-treated patients also required availability of at least 80% of INR records from the previous year. Anticoagulant therapy was chosen in routine practice before enrolment and was not assigned or modified by the protocol. The registry did not systematically record the reason for the initial anticoagulant choice, although reasons for later switching were collected. All patients provided written informed consent. Exclusion criteria were mechanical prosthetic heart valves, moderate or severe mitral stenosis, any indication for long-term VKA therapy, or participation in a clinical trial at inclusion. Requiring at least six months of prior anticoagulation may introduce prevalent-survivor bias. Moreover, VKA INR completeness was evaluated only during the year before enrolment and may not reflect control over the entire period of VKA exposure. Patients were randomly selected from each investigator’s eligible patient list. The study complied with the Declaration of Helsinki and Spanish regulations, was approved by the Clinical Research Ethics Committee of Hospital Clínico San Carlos, Madrid, Spain, and was promoted by Fundación SEMERGEN with Primary Care physicians from the SEMERGEN Research Network and relevant working groups.

Patients were recruited from 11 October 2021 to 30 January 2025. Data from AF diagnosis to study inclusion were collected retrospectively from electronic medical records and supplemented with validated questionnaires at inclusion. Variables included sociodemographic characteristics, lifestyle habits, medical history, comorbidities, anthropometric and laboratory parameters, AF characteristics, anticoagulant treatment history, DOAC dose, and clinical events during documented anticoagulant exposure. VKA control was assessed by TTR using the Rosendaal method (poor control: TTR < 65%) and the direct method (poor control: TTR < 60%). Reasons for switching or not switching from VKA to DOAC therapy were also recorded. CHA_2_DS_2_-VASc was prespecified as a descriptive variable reflecting the risk-stratification approach used during the study period. Because the 2024 ESC AF guidelines now recommend the sex-neutral CHA_2_DS_2_-VA score, RACOVIR CHA_2_DS_2_-VASc values should be interpreted descriptively and not as current treatment-eligibility criteria [10].

Patients completed validated questionnaires assessing functional autonomy with the Barthel Index and treatment satisfaction with the Anti-Clot Treatment Scale (ACTS). The Barthel Index evaluates ten activities of daily living on a 0–100 scale, with higher scores indicating greater independence [11]. The ACTS is a validated 17-item questionnaire measuring perceived anticoagulant treatment burden and benefits over the previous 4 weeks. Items 1–12 assess burden, item 13 overall negative impact on daily life, items 14–16 assess perceived benefits, and item 17 assesses overall positive impact [12]. ACTS was administered only at enrolment; therefore, cross-sectional group comparisons may reflect treatment selection and persistence.

Clinical events comprised ischemic stroke, hemorrhagic stroke, transient ischemic attack, systemic embolism, major bleeding, intracranial hemorrhage, and myocardial infarction. Ischemic events included ischemic stroke, transient ischemic attack, systemic embolism, and myocardial infarction; bleeding events included major bleeding and hemorrhagic stroke. Events were attributed to the anticoagulant exposure documented at the time of occurrence. For recurrent events of the same type within an exposure period, only the first was included in time-to-event analyses. Exposure did not necessarily match treatment at enrolment, as patients could contribute person-time to VKA exposure, DOAC exposure, or both after switching. Treatment sequences, including VKA-to-DOAC and DOAC-to-VKA switches, were recorded. Thus, event analyses were exposure-based and descriptive, not randomized or fixed-cohort comparisons.

### Statistical Analysis

Continuous variables were expressed as mean ± standard deviation (SD), while categorical variables were presented as absolute frequencies and percentages. Comparisons between patients receiving VKAs and DOACs at enrolment were performed using Student’s *t*-test or an appropriate non-parametric test for continuous variables and the chi-square test for categorical variables. Clinical events were initially analyzed according to documented anticoagulant exposure. Person-time was allocated to the corresponding treatment category (VKA or DOAC), allowing patients who switched anticoagulant strategies to contribute person-time to both groups. Crude incidence rates were calculated per 1000 person-years, and unadjusted incidence rate ratios (IRRs; DOAC/VKA) were estimated with their corresponding 95% confidence intervals (CIs). These initial analyses were considered exploratory because treatment allocation was not randomized and potential confounding factors could influence the observed associations. To account for potential confounding and treatment switching, an additional post hoc analysis was conducted using marginal structural models (MSMs) with stabilized inverse-probability-of-treatment weighting (IPTW). Treatment probabilities were estimated using multivariable logistic regression models incorporating age, sex, relevant comorbidities, bleeding risk, atrial fibrillation characteristics, calendar period, and previous anticoagulant treatment. Dynamic stabilized weights were calculated across two treatment periods, with the numerator conditioned on previous treatment and the denominator additionally conditioned on baseline covariates. To minimize the influence of extreme weights, stabilized weights were truncated at the 99th percentile. Weighted generalized linear models assuming a Poisson distribution with a logarithmic link function were subsequently fitted to estimate adjusted IRRs (DOAC/VKA) and their corresponding 95% CIs. Robust standard errors were calculated using the Huber–White sandwich estimator. An additional post hoc sensitivity analysis was performed using a propensity-score-adjusted Cox proportional hazards model restricted to 1492 patients who received only one anticoagulant strategy during follow-up, excluding patients exposed to both VKAs and DOACs. The analysis incorporated the following adjustment variables: age, sex, chronic kidney disease, previous cerebrovascular disease, heart failure, ischemic heart disease, peripheral arterial disease, bleeding risk, atrial fibrillation type, and calendar period. Adjusted hazard ratios (HRs) with 95% CIs were estimated for clinical outcomes, including stroke, major bleeding, and the composite ischemic/hemorrhagic endpoint. This complementary analysis was intended to assess the robustness of the findings in patients without documented treatment switching. All statistical tests were two-sided, with a *p*-value < 0.05 considered statistically significant. Given the observational nature of the study, the absence of randomized treatment allocation, and the post hoc nature of the adjusted analyses, results were interpreted as associations rather than causal treatment effects. Furthermore, HRs derived from Cox regression and IRRs obtained from exposure-based Poisson models represent different effect measures and were therefore interpreted separately.

## 3. Results

A total of 1901 patients with NVAF were included in the study, of whom 428 (22.5%) were receiving VKA and 1473 (77.5%) DOACs. Mean duration of treatment with VKA and DOACs was 7.6 and 3.8 years, respectively.

The study population was elderly overall, with a mean age of 78.3 ± 10.0 years. Patients receiving VKAs were significantly older than those treated with DOACs (79.7 ± 9.7 vs. 77.9 ± 10.1 years; *p* = 0.001). Sex distribution was balanced and similar between groups, with men representing approximately 52.0% of the total population. Most participants lived in urban environments (66.3%), although VKA users were more frequent from rural areas than DOAC users (24.3% vs. 13.8%; *p* < 0.001). Systolic blood pressure and heart rate were largely comparable between groups. However, VKA-treated patients had slightly lower diastolic blood pressure values (73.9 ± 10.4 vs. 75.4 ± 10.8 mmHg; *p* = 0.01).

Hypertension was the most common cardiovascular risk factor, present in 77.0% of the total cohort. It was significantly more frequent among VKA-treated patients (82.2% vs. 75.5%; *p* = 0.004). The prevalence of dyslipidemia and diabetes did not differ significantly between groups. The prevalence of chronic kidney disease, heart failure, ischemic heart disease, cerebrovascular disease, and peripheral artery disease was high in both groups, reflecting the frailty and multimorbidity of the population. However, none of these conditions differed significantly between anticoagulant strategies. Likewise, rates of dementia, sleep apnea, active cancer, and functional dependence (assessed with the Barthel index) were comparable between groups (Table 1).

Regarding AF characteristics and anticoagulant therapy, 94.5% of patients had a CHA_2_DS_2_-VASc score ≥ 2; this score is reported descriptively because current ESC guidance uses CHA_2_DS_2_-VA for thromboembolic risk assessment [10]. High bleeding risk (HAS-BLED ≥ 3) was common. Permanent AF was more frequent with VKAs (54.2%), whereas paroxysmal AF predominated with DOACs (43.8%; *p* < 0.001). Among DOAC users, apixaban was most prescribed (43.2%), followed by edoxaban (26.2%), rivaroxaban (20.5%), and dabigatran (10.0%). Standard doses were used in 75.2%, 45.3%, 73.1%, and 78.1% of apixaban, dabigatran, edoxaban, and rivaroxaban users, respectively; corresponding reduced doses were 24.8%, 54.7%, 26.9%, and 21.9%. These data reflect prescribed doses only and did not establish their appropriateness based on age, renal function, body weight, or drug-specific criteria. Acenocoumarol was used by 97.9% of VKA users. VKA control was suboptimal: 43.8% had poor INR control by the direct method and 41.0% by Rosendaal (Table 2). Overall, 395 patients (20.8%) received both VKA and DOAC therapy during follow-up: 372 switched from VKA to DOAC and 23 from DOAC to VKA. Recorded reasons for VKA-to-DOAC switching included poor INR control (23.2%) and difficulty accessing INR monitoring (9.4%).

Treatment satisfaction was assessed using the ACTS questionnaire. Patients receiving DOACs reported greater satisfaction with therapy than those on VKAs. DOAC users more frequently described the treatment as having a positive impact and less frequently reported negative effects on daily life (*p* < 0.001). They also expressed higher satisfaction with treatment overall (Table 3).

During the study period, 539 clinical events were recorded in 470 patients (Table 4), of which 89.0% were ischemic and 11.0% hemorrhagic. Overall, 395 patients switched anticoagulant strategies during follow-up and consequently contributed person-time to both treatment groups. Clinical events and exposure time were assigned to the anticoagulant strategy documented at the time of the event or during the corresponding treatment interval. In the unadjusted analysis, crude incidence rate ratios (IRRs; DOAC/VKA) were 1.75 (95% CI 1.05–2.92; *p* = 0.040) for stroke, 1.78 (95% CI 1.06–3.00; *p* = 0.040) for ischemic stroke, 1.64 (95% CI 0.97–2.79; *p* = 0.080) for myocardial infarction, and 3.68 (95% CI 1.81–7.46; *p* < 0.001) for major bleeding (Table 5). These crude comparisons were exploratory and did not account for differences in baseline clinical characteristics, treatment selection, or other potential confounding factors.

To further investigate the associations between anticoagulant exposure and clinical outcomes, an additional post hoc analysis was performed using marginal structural models with stabilized inverse-probability weighting (Table 6). After adjustment, no statistically significant associations were observed for stroke (IRR 1.627; 95% CI 0.785–3.375; *p* = 0.191), ischemic stroke (IRR 1.537; 95% CI 0.738–3.201; *p* = 0.251), systemic embolism (IRR 0.822; 95% CI 0.151–4.480; *p* = 0.820), or myocardial infarction (IRR 1.035; 95% CI 0.566–1.892; *p* = 0.910). Transient ischemic attack showed a lower incidence during DOAC exposure (IRR 0.441; 95% CI 0.195–0.998; *p* = 0.049). In contrast, DOAC exposure was associated with a higher incidence of major bleeding (IRR 6.725; 95% CI 2.106–21.473; *p* = 0.001) and the combined ischemic or hemorrhagic endpoint (IRR 1.600; 95% CI 1.096–2.335; *p* = 0.015). No statistically significant association was observed for the composite ischemic endpoint (IRR 1.099; 95% CI 0.729–1.657; *p* = 0.652). The estimate for any hemorrhagic event was identical to that for major bleeding (IRR 6.725; 95% CI 2.106–21.473; *p* = 0.001).

An additional post hoc sensitivity analysis was conducted using a propensity-score-adjusted Cox proportional hazards model restricted to 1492 patients who received a single anticoagulant strategy throughout the observation period (Table 7). Consistent with the marginal structural model, no statistically significant associations were observed for stroke (HR 2.577; 95% CI 0.930–7.144; *p* = 0.069), ischemic stroke (HR 2.345; 95% CI 0.837–6.564; *p* = 0.105), transient ischemic attack (HR 0.645; 95% CI 0.209–1.993; *p* = 0.446), systemic embolism (HR 0.847; 95% CI 0.130–5.502; *p* = 0.861), or myocardial infarction (HR 0.873; 95% CI 0.417–1.824; *p* = 0.717). However, DOAC exposure remained associated with a higher hazard of major bleeding (HR 8.298; 95% CI 3.180–21.649; *p* < 0.001) and the combined ischemic or hemorrhagic endpoint (HR 2.004; 95% CI 1.268–3.167; *p* = 0.003). No statistically significant association was observed for any ischemic event (HR 1.135; 95% CI 0.681–1.891; *p* = 0.628), whereas the estimate for any hemorrhagic event was identical to that for major bleeding (HR 8.298; 95% CI 3.180–21.649; *p* < 0.001).

Across both adjusted analyses, DOAC exposure was associated with higher estimates for major bleeding and the combined ischemic or hemorrhagic endpoint, whereas the association with stroke did not reach statistical significance. Nevertheless, the two models differed in their analytical populations and effect measures, and their estimates should not be considered directly interchangeable. Given the observational design, post hoc analyses, and potential residual confounding, these findings should be interpreted as exploratory associations rather than evidence of causal differences in anticoagulant effectiveness or safety.

## 4. Discussion

In this large real-world registry of patients with NVAF managed in Spanish Primary Care, DOACs predominated at enrolment, whereas nearly 40% of VKA-treated patients had suboptimal INR control. DOAC users reported greater treatment satisfaction and less interference with daily life. Longitudinal exposure was complex: one in five patients received both VKA and DOAC therapy during follow-up. Crude exposure-based event analyses differed by treatment exposure, but these findings are exploratory and cannot establish comparative effectiveness or safety because treatment was not randomized and groups differed in exposure duration, clinical profile, dosing patterns, and switching history. A supplementary inverse-probability-weighted marginal structural model attenuated the crude stroke association, although the estimated association for major bleeding remained elevated with a wide confidence interval. The discordance with randomized evidence, potential residual and time-varying confounding, and uncertain outcome ascertainment preclude causal interpretation.

The clinical profile observed in RACOVIR aligns with findings from recent Spanish and international AF registries. Patients were mainly older adults with multiple comorbidities and high thromboembolic risk, particularly hypertension, diabetes mellitus, chronic kidney disease, and heart failure. This pattern is consistent with contemporary Spanish real-world cohorts of anticoagulated patients with AF. Similar results have been reported in the AFIRMA study and the SULTAN registry, both of which showed that anticoagulated patients with AF in Spain are becoming older, frailer, and more clinically complex [13,14]. In AFIRMA, hypertension, diabetes, heart failure, and renal disease were reported in 76.3%, 48.0%, 42.2%, and 18.7% of patients, respectively, and these conditions were more common among VKA users [13]. Likewise, SULTAN included a predominantly elderly population (mean age 73.6 years) with high CHA_2_DS_2_-VASc scores and a substantial burden of comorbidity [14]. Other Spanish observational studies, including FANTASIIA and ESPARTA, further support the growing frailty and clinical complexity of anticoagulated AF populations in routine practice [15,16]. In our cohort, VKA-treated patients were older and more likely to live in rural areas. This may reflect historical prescribing patterns, more limited access to specialized anticoagulation care, and slower uptake of DOACs in underserved settings. Spanish real-world studies have also shown marked geographic and healthcare-level variation in anticoagulant initiation and prescribing. In particular, García-Sempere et al. reported substantial regional differences in oral anticoagulant initiation among patients with AF in Spain, suggesting that healthcare organization and access may influence treatment choice [17]. Permanent AF was more frequent among VKA users, whereas paroxysmal AF predominated in DOAC-treated patients, consistent with previous Southern European observational studies comparing both treatment groups in routine practice [13,14,15,16]. Overall, these findings may support the external validity of RACOVIR and suggest that its population is representative of patients with NVAF managed in contemporary Spanish clinical practice.

Earlier Spanish and international registries showed that up to 20–30% of patients with AF and high thromboembolic risk were treated with antiplatelet therapy alone or received no antithrombotic treatment [18,19,20,21]. Encouragingly, this pattern has declined over time, largely because of increased use of anticoagulants, particularly DOACs, accompanied by declining rates of AF-related stroke at the population level [22,23,24]. In Spain, DOAC use has increased steadily in recent years and has largely replaced VKAs in newly anticoagulated patients with NVAF. This shift likely reflects accumulating evidence on the efficacy and safety of DOACs, updated guideline recommendations, and greater physician familiarity with these agents. This progressive shift toward DOACs has also been observed in other thromboembolic conditions, particularly deep vein thrombosis, reflecting their more predictable anticoagulant effects, simplified management, and reduced need for routine laboratory monitoring [25]. Consistent with this trend, about three quarters of patients in RACOVIR were receiving DOACs, whereas one quarter were treated with VKAs. Nevertheless, DOAC prescription rates in Spain have historically remained lower than in other European countries, partly because of regional administrative restrictions and reimbursement policies [8,22,23,24].

Suboptimal anticoagulation control remains a significant challenge among VKA-treated patients in Spain. In RACOVIR, approximately 40% of patients receiving VKAs exhibited inadequate INR control, as assessed by both the Rosendaal and direct TTR methods, consistent with previous Spanish registries reporting poor anticoagulation control in nearly half of VKA-treated patients, including recent cohorts [26,27,28,29]. Given the established association between inadequate INR control and increased thromboembolic and bleeding risks, poor anticoagulation quality remains an important reason for switching from VKAs to DOACs [30]. However, the interpretation of comparative outcomes between anticoagulant strategies warrants caution. Although the additional propensity-score-adjusted Cox sensitivity analysis provides complementary information, it excludes patients who switched treatments, potentially introducing selection bias and limiting generalizability. Furthermore, the absence of propensity-score diagnostics, proportional-hazards assessments, and endpoint-specific event counts, together with differences in analytical populations and effect measures (HR versus IRR) compared with the weighted analysis, limits the robustness and comparability of these findings. Therefore, comparative treatment outcomes should be interpreted as exploratory rather than as evidence of differences in effectiveness or safety. Nevertheless, these findings underscore the need to improve INR control among VKA-treated patients to optimize anticoagulation management and potentially reduce the risk of thromboembolic and bleeding complications.

Treatment satisfaction is a key component of long-term anticoagulation management, as it may influence adherence, treatment persistence, and clinical outcomes. In RACOVIR, patients receiving DOACs reported greater satisfaction, lower treatment burden, and less interference with daily activities than those treated with VKAs. These findings are consistent with the simpler management of DOACs, including fixed dosing, no routine INR monitoring, and fewer food and drug interactions. Previous studies using validated patient-reported outcome measures, including the ACTS questionnaire, have similarly demonstrated greater convenience and satisfaction with DOACs compared with VKAs, particularly relevant in elderly, polymedicated patients with AF requiring lifelong anticoagulation [12,31]. From a clinical outcomes perspective, an additional propensity-score-adjusted Cox sensitivity analysis involving 1492 patients receiving a single anticoagulant strategy showed results broadly consistent in direction with the weighted analysis for major bleeding and the combined ischemic/hemorrhagic endpoint, whereas the association with stroke remained statistically inconclusive. However, given the observational nature of the study and the methodological limitations of these analyses, neither approach establishes a causal relationship between anticoagulant strategy and clinical outcomes. Overall, these findings highlight the importance of considering patient-reported satisfaction and treatment burden alongside clinical effectiveness and safety when individualizing long-term anticoagulation strategies.

Crude exposure-based incidence rates during DOAC exposure were higher than during VKA exposure for some outcomes and should be interpreted cautiously. Although these findings contrast with randomized trials and meta-analyses showing comparable efficacy and generally favorable safety of DOACs versus VKAs [32,33], RACOVIR was not designed to assess causal comparative effectiveness. VKA exposure was substantially longer, 20.8% of patients received both strategies, and switching to DOACs often followed poor INR control or treatment instability, raising the possibility of confounding by indication and channeling of higher-risk patients. Evidence specifically addressing switching in frail older patients is heterogeneous. In the randomized FRAIL-AF trial, switching clinically stable VKA-treated patients to DOACs increased bleeding without reducing thromboembolic events [34]. In contrast, the COMBINE-AF substudy reported lower risks of stroke or systemic embolism, fatal and intracranial bleeding, and death after switching to standard-dose DOACs, although gastrointestinal bleeding increased and major bleeding was similar [35]. A 2026 Korean nationwide time-varying analysis found higher risks of adverse outcomes after switching frail older patients stably treated with warfarin [36]. Moreover, a recent systematic review and meta-analysis found no significant overall reductions in thromboembolism, major bleeding, mortality, or composite outcomes with switching [37], reinforcing the need for individualized decisions rather than routine switching in stable patients [38]. These apparently divergent findings likely reflect differences in populations, frailty definitions, anticoagulation quality, switching strategies, and study designs, and are particularly relevant to the 20.8% of RACOVIR participants exposed to both anticoagulant strategies. DOAC dose selection may also have contributed, as reduced doses were frequent, although appropriateness could not be assessed from the available aggregate data. Thus, the unadjusted IRRs in Table 5 should be considered hypothesis-generating descriptive signals, not evidence that one strategy is more effective or safer than another [39].

From a Primary Care perspective, these findings identify several modifiable aspects of anticoagulation management. The high proportion of VKA-treated patients with inadequate TTR supports regular review of anticoagulation quality and consideration of alternatives when control remains poor. Frequent reduced DOAC dosing highlights the need to reassess dose against drug-specific criteria, particularly renal function, age, and body weight. The 20.8% exposure to both strategies indicates that switching is common and should be considered in outcome interpretation and medication reconciliation. Greater satisfaction with DOACs also suggests that patient burden and preferences should inform shared decision-making alongside thromboembolic and bleeding risk.

This study has several limitations. First, its observational design and retrospective longitudinal data collection preclude causal inference, while the lack of randomization and systematically recorded reasons for initial treatment selection introduce potential confounding by indication and selection bias. Furthermore, the six-month eligibility criterion may have introduced prevalent-user and survivor bias. Second, treatment exposure differed substantially between VKAs and DOACs, and switching between anticoagulant strategies was frequent. Although clinical events were assigned according to treatment exposure, the absence of an adjusted time-varying treatment model limits the interpretation of crude incidence rate ratios (IRRs), which may be influenced by differences in baseline risk, renal function, treatment duration, dose selection, adherence, switching patterns, and other measured or unmeasured confounders. Third, although DOAC prescriptions and doses were recorded, the appropriateness of dose reduction could not be reliably assessed for all agents. Additional information on treatment adherence, temporary interruptions, socioeconomic status, comprehensive frailty measures, and detailed switching chronology was unavailable. Similarly, additional patient-level analyses evaluating TTR < 70%, DOAC dose appropriateness, and adjusted or switcher-stratified ACTS scores could not be performed. Fourth, the post hoc propensity-score-weighted analysis was limited by the unavailability of weighting diagnostics, covariate balance assessments, detailed exposure episode definitions, exposure-specific person-time, and comprehensive information on missing-data handling. The additional propensity-score-adjusted Cox sensitivity analysis, restricted to patients receiving a single anticoagulant strategy, also excluded switchers and lacked complete model diagnostics, further limiting the robustness and generalizability of the adjusted estimates. Consequently, both adjusted analyses should be considered exploratory, given the potential for residual confounding, model misspecification, and outcome misclassification. Finally, CHA_2_DS_2_-VASc was collected according to the prespecified study protocol and is reported for historical descriptive purposes, although current ESC guidelines recommend CHA_2_DS_2_-VA [10]. While RACOVIR provides valuable real-world evidence from Spanish Primary Care, its findings may not be fully generalizable to hospital-based populations or healthcare systems with different anticoagulation management practices.

## 5. Conclusions

In conclusion, RACOVIR offers a real-world overview of oral anticoagulation in patients with NVAF followed in Spanish Primary Care. DOACs predominated at enrolment and were associated with greater patient-reported satisfaction, whereas suboptimal INR control remained frequent among VKA-treated patients. Switching between strategies and reduced DOAC dosing were common, underscoring the need for regular review of treatment quality, dose appropriateness, and patient preferences. Crude exposure-based event differences should not be interpreted as comparative effectiveness or safety estimates because of the observational design, unequal exposure duration, treatment switching, and residual confounding. Overall, these findings support quality-improvement initiatives and future patient-level analyses using adjusted, time-varying methods. The supplementary weighted estimates do not eliminate confounding by indication and cannot establish comparative effectiveness or safety.

## Figures and Tables

**Table 1:** Baseline clinical characteristics of the study population.

	VKA (*n* = 428; 22.5%)	DOACs (*n* = 1473; 77.5%)	Total n (%) (*n* = 1901; 100%)	*p*
Biodemographic data				
Age, years	79.7 ± 9.7	77.9 ± 10.1	78.3 ± 10.0	0.001
≤70 years, *n* (%)	71 (16.6)	311 (21.1)	382 (20.1)	
71–80 years, *n* (%)	132 (30.8)	523 (35.5)	655 (34.5)	0.003
≥81 years, *n* (%)	225 (52.6)	639 (43.4)	864 (45.4)	
Sex, male, *n* (%)	228 (53.3)	761 (51.7)	989 (52.0)	0.558
Habitat, *n* (%)				
Rural	104 (24.3)	203 (13.8)	307 (16.2)	
Semi-urban	65 (15.2)	268 (18.2)	333 (17.5)	<0.001
Urban	259 (60.5)	1002 (68.0)	1261 (66.3)	
Autonomous community, *n* (%)				
Andalusia	5 (1.2)	58 (3.9)	63 (3.3)	
Aragon	10 (2.3)	16 (1.1)	26 (1.4)	
Asturias	16 (3.7)	16 (1.1)	32 (1.7)	
Balearic Islands	1 (0.2)	11 (0.7)	12 (0.6)	
Canary Islands	15 (3.5)	109 (7.4)	124 (6.5)	
Cantabria	0 (0.0)	0 (0.0)	0 (0.0)	
Castilla y Leon	131 (30.6)	713 (48.4)	844 (44.4)	
Castilla-La Mancha	8 (1.9)	12 (0.8)	20 (1.1)	<0.001
Catalonia	10 (2.3)	22 (1.5)	32 (1.7)	
Valencia	19 (4.4)	82 (5.6)	101 (5.3)	
Extremadura	80 (18.7)	211 (14.3)	291 (15.3)	
Galicia	7 (1.6)	19 (1.3)	26 (1.4)	
Madrid	116 (27.1)	193 (13.1)	309 (16.3)	
Murcia	0 (0.0)	0 (0.0)	0 (0.0)	
Navarra	9 (2.1)	11 (0.7)	20 (1.1)	
Basque Country	0 (0.0)	0 (0.0)	0 (0.0)	
La Rioja	1 (0.2)	0 (0.0)	1 (0.1)	
Physical examination				
Systolic blood pressure, mmHg	130.7 ± 15.8	130.5 ± 15.4	130.6 ± 15.5	0.877
Diastolic blood pressure, mmHg	73.9 ± 10.4	75.4 ± 10.8	75.1 ± 10.7	0.01
Heart rate, bpm	72.2 ± 12.7	72.7 ± 12.5	72.6 ± 12.5	0.484
Body mass index, kg/m^2^	29.3 ± 5.7	28.8 ± 5.0	28.9 ± 5.2	0.043
Body weight, kg	78.6 ± 17.1	77.2 ± 15.4	77.5 ± 15.8	0.086
Waist circumference, cm	100.7 ± 15.4	98.3 ± 15.5	98.8 ± 15.5	0.005
Lifestyle Habits				
Physical activity, *n* (%)				
Sedentary	248 (57.9)	719 (48.8)	967 (50.9)	
Low-moderate	137 (32.0)	590 (40.1)	727 (38.2)	
Moderate	36 (8.4)	141 (9.6)	177 (9.3)	0.015
Regular sport practice	6 (1.4)	21 (1.4)	27 (1.4)	
Alcohol abuse, %	27 (6.3)	103 (7.0)	130 (6.8)	0.619
Smoking status, *n* (%)				
Current smoker	21 (4.9)	102 (6.9)	123 (6.5)	
Non-smoker	273 (63.8)	932 (63.3)	1205 (63.4)	0.496
Former smoker	126 (29.4)	413 (28.1)	539 (28.4)	
Cardiovascular risk factors				
Hypertension, *n* (%)	352 (82.2)	1112 (75.5)	1464 (77.0)	0.004
Dyslipidemia, *n* (%)	282 (65.9)	1024 (69.6)	1306 (68.7)	0.149
Diabetes mellitus, *n* (%)	140 (32.7)	503 (34.2)	643 (33.8)	0.574
Cardiovascular disease				
Chronic kidney disease, *n* (%)	139 (32.5)	427 (29.0)	566 (29.8)	0.165
Heart failure, *n* (%)	112 (26.2)	397 (27.0)	509 (26.8)	0.747
Ischemic heart disease, *n* (%)	73 (17.1)	247 (16.8)	320 (16.8)	0.889
Cerebrovascular disease, *n* (%)	36 (8.4)	168 (11.4)	204 (10.7)	0.078
Peripheral artery disease, *n* (%)	47 (11.0)	119 (8.1)	166 (8.7)	0.061
Other comorbidities				
Dementia, *n* (%)	36 (8.4)	107 (7.3)	143 (7.5)	0.428
Sleep apnea, *n* (%)	28 (6.5)	103 (7.0)	131 (6.9)	0.746
Active cancer, *n* (%)	19 (4.4)	85 (5.8)	104 (5.5)	0.286
Barthel	85.1 ± 24.3	85.6 ± 22.8	85.4 ± 23.2	0.71
Biochemical parameters				
Hemoglobin, g/L	14.1 ± 1.9	13.8 ± 1.9	13.9 ± 1.9	0.017
Glucose, mg/dL	107.2 ± 29.4	108.3 ± 29.4	108.1 ± 29.4	0.484
HbA1c, %	6.2 ± 0.8	6.2 ± 0.9	6.2 ± 0.9	0.767
Creatinine, mg/dL	1.07 ± 0.5	1.05 ± 0.4	1.06 ± 0.4	0.419
eGFR, mL/min/1.73 m^2^	64.1 ± 18.8	65.1 ± 19.1	64.8 ± 19.1	0.392
UACR, mg/g	25.6 ± 33.9	20.9 ± 29.1	22.0 ± 30.4	0.08
NT-proBNP, pg/mL	1302.9 ± 1416.6	1197.4 ± 1241.1	1218.7 ± 1277.4	0.487
ALT, UI/L	20.2 ± 11.7	22.2 ± 14.3	21.7 ± 13.8	0.016
Total cholesterol, mg/dL	163.2 ± 34.2	167.0 ± 41.9	166.1 ± 40.4	0.102
HDL cholesterol, mg/dL	51.9 ± 14.2	52.6 ± 13.6	53.4 ± 13.7	0.42
LDL cholesterol, mg/dL	90.7 ± 32.0	94.3 ± 35.3	93.5 ± 34.7	0.105
Triglycerides, mg/dL	117.3 ± 54.3	114.0 ± 52.9	114.8 ± 53.2	0.294

ALT: alanine aminotransferase; DOACs: direct oral anticoagulants; HbA1c: glycated hemoglobin; HDL: high-density lipoprotein; LDL: low-density lipoprotein; NT-proBNP: N-terminal pro-B-type natriuretic peptide; UACR: urine albumin-to-creatinine ratio; VKA: vitamin K antagonist.

**Table 2:** Characteristics of AF and anticoagulant treatment.

Variable	VKA (*n* = 428; 22.5%)	DOACs (*n* = 1473; 77.5%)	Total (*n* = 1901; 100%)	*p*
CHA2DS2-VASc, *n* (%)				
0	2 (0.6)	17 (1.4)	19 (1.2)	
1	8 (2.2)	78 (6.3)	86 (5.4)	0.013
≥2	418 (97.2)	1378 (93.3)	1796 (94.5)	
HAS-BLED ≥ 3, *n* (%)	116 (3.4)	325 (28.9)	441 (30.2)	0.04
Type of AF, *n* (%)				
Paroxysmal	138 (32.2)	645 (43.8)	783 (41.2)	
Persistent	58 (13.6)	241 (16.4)	299 (15.7)	<0.001
Permanent	232 (54.2)	587 (39.9)	819 (43.1)	
Type of DOACs, *n* (%)				
Apixaban		637 (43.2)		
Dabigatran	-	148 (10.0)	-	-
Edoxaban		386 (26.2)		
Rivaroxaban		302 (20.5)		
Dose of DOACs				
Apixaban 5 mg/12 h (vs. total apixaban)		479 (75.2)		-
Dabigatran 150 mg/12 h (vs. total dabigatran)	-	67 (45.3)	-	
Edoxaban 60 mg/day (vs. total edoxaban)		282 (73.1)		
Rivaroxaban 20 mg/day (vs. total rivaroxaban)		236 (78.1)		
Type of VKA, *n* (%)				
Acenocoumarol	419 (97.9)	-	-	-
Warfarin	9 (2.1)			
INR control in VKA-treated patients				
TTR (direct method), %	61.8 ± 17.8			
TTR (Rosendaal method), %	68.1 ± 17.8	-	-	-
Poor control by direct method (<60%), *n* (%)	187 (43.8)			
Poor control by Rosendaal method (<65%), *n* (%)	175 (41.0)			
Previous AF ablation, *n* (%)	14 (3.3)	90 (6.1)	104 (5.5)	0.013
Previous cardioversion, *n* (%)	40 (9.3)	161 (10.9)	201 (10.6)	0.194

DOACs: direct oral anticoagulants; TTR: time in the therapeutic range; VKA: vitamin K antagonist.

**Table 3:** ACTS questionnaire. Satisfaction with the treatment received.

		VKA (*n* = 428; 22.5%)	DOACs (*n* = 1473; 77.5%)	Total (*n* = 1901; 100%)	*p*
Benefits with treatment—health	Not at all, *n* (%)	3 (0.7)	11 (0.8)	14 (0.7)	0.074
	A little bit, *n* (%)	21 (5.1)	56 (3.8)	77 (4.1)	
	Moderately, *n* (%)	74 (17.9)	188 (12.9)	262 (14.0)	
	Quite, *n* (%)	197 (47.6)	755 (51.7)	952 (50.8)	
	A lot, *n* (%)	119 (28.7)	451 (30.9)	570 (30.4)	
Benefits with treatment—satisfaction	Not at all, *n* (%)	6 (1.4)	12 (0.8)	18 (1.0)	<0.001
	A little bit, *n* (%)	28 (6.8)	49 (3.4)	77 (4.1)	
	Moderately, *n* (%)	91 (22.0)	186 (12.7)	277 (14.8)	
	Quite, *n* (%)	204 (49.3)	770 (52.7)	974 (51.9)	
	A lot, *n* (%)	85 (20.5)	444 (30.4)	529 (28.2)	
Benefits with treatment—calm	Not at all, *n* (%)	6 (1.4)	11 (0.8)	17 (0.9)	0.127
	A little bit, *n* (%)	20 (4.8)	49 (3.4)	69 (3.7)	
	Moderately, *n* (%)	70 (16.9)	239 (16.4)	309 (16.5)	
	Quite, *n* (%)	224 (54.1)	756 (51.7)	980 (52.3)	
	A lot, *n* (%)	94 (22.7)	406 (27.8)	500 (26.7)	
Negative impact in daily life	Not at all, *n* (%)	166 (40.1)	845 (57.8)	1011 (53.9)	<0.001
	A little bit, *n* (%)	168 (40.6)	478 (32.7)	646 (34.5)	
	Moderately, *n* (%)	56 (13.5)	93 (6.4)	149 (7.9)	
	Quite, *n* (%)	19 (4.6)	32 (2.2)	51 (2.7)	
	A lot, *n* (%)	5 (1.2)	13 (0.9)	18 (1.0)	
Positive impact	Not at all, *n* (%)	10 (2.4)	20 (1.4)	30 (1.6)	<0.001
	A little bit, *n* (%)	27 (6.5)	59 (4.0)	86 (4.6)	
	Moderately, *n* (%)	95 (22.9)	197 (13.5)	292 (15.6)	
	Quite, *n* (%)	205 (49.5)	751 (51.4)	956 (51.0)	
	A lot, *n* (%)	77 (18.6)	434 (29.7)	511 (27.3)	

**Table 4:** Clinical events.

Event Type	*n* (%)	Number of Events per Patient	Number of Patients *n* (%)
Myocardial infarction	224 (41.6)	1 event	411 (87.4)
Stroke	159 (29.5)	2 events	52 (11.1)
Transient ischemic attack	72 (13.4)	3 events	4 (0.9)
Major bleeding	54 (10.0)	4 events	3 (0.6)
Systemic embolism	30 (5.6)		
Any ischemic event	480 (89.0)		
Any bleeding event	59 (11.0)		

Any ischemic event: ischemic stroke, transient ischemic attack, systemic embolism, myocardial infarction. Any bleeding event: major bleeding, hemorrhagic stroke.

**Table 5:** Risk of events during treatment with oral anticoagulants.

	Anticoagulant	*N*	Events, *n* (%)	*P*_proportions_	Mean Follow-Up (Years)	Person-Years Follow-Up	Incidence Rate per 1000 Person-Years	Incidence Rate Ratio DOAC/VKA (95%CI)	*P*_incidence rate_
Stroke	VKA	819	24 (2.93)	0.71	7.55	6183.16	3.88	1.75 (1.05–2.92)	0.04
	DOACs	1477	38 (2.57)		3.78	5584.71	6.80		
Ischemic stroke	VKA	819	23 (2.81)	0.76	7.55	6183.18	3.72	1.78 (1.06–3.00)	0.04
	DOACs	1477	37 (2.51)		3.78	5585.00	6.63		
Transient ischemic attack	VKA	819	16 (1.95)	0.03	7.61	6234.94	2.57	0.83 (0.39–1.75)	0.76
	DOACs	1477	12 (0.81)		3.83	5654.63	2.12		
Systemic embolism	VKA	819	4 (0.49)	0.63	7.70	6305.45	0.63	1.11 (0.21–5.97)	0.05
	DOACs	1477	4 (0.27)		3.84	5672.98	0.71		
Myocardial infarction	VKA	819	23 (2.81)	0.54	7.57	6195.64	3.71	1.64 (0.97–2.79)	0.08
	DOACs	1477	34 (2.30)		3.77	5572.00	6.10		
Major bleeding	VKA	819	10 (1.22)	0.12	7.70	6302.65	1.59	3.68 (1.81–7.46)	0.0002
	DOACs	1477	33 (2.23)		3.83	5657.16	5.83		
Any ischemic event	VKA	819	63 (7.69)	0.09	7.31	5983.01	10.53	1.50 (1.08–2.07)	0.02
	DOACs	1477	86 (5.82)		3.69	5453.41	15.77		
Any hemorrhagic event	VKA	819	11 (1.34)	0.15	7.69	6295.53	1.75	3.34 (1.69–6.61)	0.0004
	DOACs	1477	33 (2.23)		3.83	5657.16	5.83		
Any ischemic or hemorrhagic event	VKA	819	72 (8.79)	0.50	7.29	5969.53	12.06	1.80 (1.34–2.41)	0.0001
	DOACs	1477	118 (7.99)		3.68	5433.78	21.72		

DOACs: direct oral anticoagulants; VKA: vitamin K antagonist.

**Table 6:** Exploratory weighted marginal structural model results (DOAC [N = 1560] versus VKA [N = 467]).

Outcome	Adjusted IRR	95% Confidence Interval	*p* Value
Stroke	1.627	0.785–3.375	0.191
Ischemic stroke	1.537	0.738–3.201	0.251
Transient ischemic attack	0.441	0.195–0.998	0.049
Systemic embolism	0.822	0.151–4.480	0.820
Myocardial infarction	1.035	0.566–1.892	0.910
Major bleeding	6.725	2.106–21.473	0.001
Any ischemic event	1.099	0.729–1.657	0.652
Any hemorrhagic event	6.725	2.106–21.473	0.001
Any ischemic or hemorrhagic event	1.600	1.096–2.335	0.015

Adjustment variables: age, sex, chronic kidney disease, previous cerebrovascular disease, heart failure, ischemic heart disease, peripheral arterial disease, bleeding risk, atrial fibrillation type, and calendar period. DOACs: direct oral anticoagulants; VKA: vitamin K antagonist.

**Table 7:** Post hoc propensity-score-adjusted Cox sensitivity analysis restricted to 1492 patients exposed to only one anticoagulant strategy (DOAC versus VKA; VKA reference).

Outcome	Adjusted HR	95% Confidence Interval	*p* Value
Stroke	2.577	0.930–7.144	0.069
Ischemic stroke	2.345	0.837–6.564	0.105
Transient ischemic attack	0.645	0.209–1.993	0.446
Systemic embolism	0.847	0.130–5.502	0.861
Major bleeding	8.298	3.180–21.649	<0.001
Myocardial infarction	0.873	0.417–1.824	0.717
Any ischemic event	1.135	0.681–1.891	0.628
Any hemorrhagic event	8.298	3.180–21.649	<0.001
Any ischemic or hemorrhagic event	2.004	1.268–3.167	0.003

Adjustment variables: age, sex, chronic kidney disease, previous cerebrovascular disease, heart failure, ischemic heart disease, peripheral arterial disease, bleeding risk, atrial fibrillation type, and calendar period. DOACs: direct oral anticoagulants; VKA: vitamin K antagonist.

## Data Availability

The minimal data set is available at repository RACOVIR via https://semergen.es/?seccion=investigacion&subSeccion=repositorioRacovir (accessed on 13 February 2025).

## Abbreviations

The following abbreviations are used in this manuscript:

ACTS	Anti-Clot Treatment Scale
AF	Atrial fibrillation
DOAC	Direct Acting Oral Anticoagulant
INR	International Normalized Ratio
NVAF	Non-Valvular Atrial fibrillation
TTR	Time in Therapeutic Range
VKA	Vitamin K Antagonists
